# Sleep Paralysis in Brazilian Folklore and Other Cultures: A Brief Review

**DOI:** 10.3389/fpsyg.2016.01294

**Published:** 2016-09-07

**Authors:** José F. R. de Sá, Sérgio A. Mota-Rolim

**Affiliations:** ^1^Graduate Program, Jungian Institute of BahiaSalvador, Brazil; ^2^Brain Institute, Federal University of Rio Grande do NorteNatal, Brazil; ^3^Department of Physiology, Federal University of Rio Grande do NorteNatal, Brazil; ^4^Onofre Lopes University Hospital, Federal University of Rio Grande do NorteNatal, Brazil

**Keywords:** sleep paralysis, Pisadeira tale, ethnopsychology, transcultural psychiatry, REM sleep, hypnopompic hallucinations

## Abstract

Sleep paralysis (SP) is a dissociative state that occurs mainly during awakening. SP is characterized by altered motor, perceptual, emotional and cognitive functions, such as inability to perform voluntary movements, visual hallucinations, feelings of chest pressure, delusions about a frightening presence and, in some cases, fear of impending death. Most people experience SP rarely, but typically when sleeping in supine position; however, SP is considered a disease (parasomnia) when recurrent and/or associated to emotional burden. Interestingly, throughout human history, different peoples interpreted SP under a supernatural view. For example, Canadian Eskimos attribute SP to spells of shamans, who hinder the ability to move, and provoke hallucinations of a shapeless presence. In the Japanese tradition, SP is due to a vengeful spirit who suffocates his enemies while sleeping. In Nigerian culture, a female demon attacks during dreaming and provokes paralysis. A modern manifestation of SP is the report of “alien abductions”, experienced as inability to move during awakening associated with visual hallucinations of aliens. In all, SP is a significant example of how a specific biological phenomenon can be interpreted and shaped by different cultural contexts. In order to further explore the ethnopsychology of SP, in this review we present the “Pisadeira”, a character of Brazilian folklore originated in the country’s Southeast, but also found in other regions with variant names. Pisadeira is described as a crone with long fingernails who lurks on roofs at night and tramples on the chest of those who sleep on a full stomach with the belly up. This legend is mentioned in many anthropological accounts; however, we found no comprehensive reference on the Pisadeira from the perspective of sleep science. Here, we aim to fill this gap. We first review the neuropsychological aspects of SP, and then present the folk tale of the Pisadeira. Finally, we summarize the many historical and artistic manifestations of SP in different cultures, emphasizing the similarities and differences with the Pisadeira.

## Introduction

Sleep paralysis (SP) is a dissociative state in which an individual, upon going to sleep or waking up, is unable to move (Mahowald et al., 2011). SP is accompanied by frightening, and often fantastic, hallucinations and delusions (Dahlitz and Parkes, 1993), thus different societies interpreted it under a supernatural or metaphysical perspective (Hinton et al., 2005). Interestingly, there is a similarity between these manifestations and the description of nocturnal assaults by the *Pisadeira*, a folk figure typical of Southeastern Brazil, but also found in other regions (Cascudo, 2012). We performed surveys using Google Scholar, as well as the Bireme and Scielo databases, but could not find comprehensive studies wherein the various neuropsychological and sociocultural aspects of SP are described and compared with the Pisadeira tale. The objective of this review is to fill this gap.

## Sleep Paralysis: Definition, Epidemiology, Neuropsychology, and Clinical Picture

The term “sleep paralysis” was coined by Wilson (1928). SP is considered a disease when recurrent and disturbing, according to the International Classification of Sleep Disorder – 3rd edition (ICSD-3) (American Academy of Sleep Medicine [AASM], 2014). SP is classified as a parasomnia and characterized by unusual behavior or abnormal physiological events that occur during the transition between sleep and wakefulness. SP episodes are generally accompanied by intense anxiety, inability to perform voluntary movements (even to scream or cry out for help), and, in some cases, fear of impending death (Sharpless et al., 2010; Jalal and Hinton, 2013). SP occurs most commonly in women (Pires et al., 2007; Sharpless and Barber, 2011) and when the body is in supine position (Chilcott et al., 1998; Sharpless et al., 2010). The prevalence of SP among the general population is controversial, ranging from 5 to 62% (Dahlitz and Parkes, 1993). In a sample of nearly two thousand Canadian university students, Burgess et al. (1995) found that 21% of these subjects experienced SP; however, in this sample there was no significant inter-gender difference. In a systematic review, Sharpless and Barber (2011) observed that the prevalence rate of at least one episode of SP in lifetime for the general population, student samples, and psychiatric patients was 7.6, 28.3, and 31.9%, respectively.

Sleep paralysis is associated with either hypnagogic hallucinations (that occur at the onset of sleep) or hypnopompic ones (when waking up) (Dahlitz and Parkes, 1993). Cheyne et al. (1999) grouped the hallucinations associated with SP into three types: (a) “Intruder”; (b) “Unusual Bodily Experiences”; (c) “Incubus.” The “Intruder” type is characterized by a feeling of fear or an unpleasant presence, accompanied by auditory and visual hallucinations. The “Unusual Bodily Experiences” involve hovering sensations and out-of-body experiences, in which individuals see the own body from an external perspective, and interpret as if they have left their physical body (Blackmore and Parker, 2002; Blanke et al., 2004; de Sá and Mota-Rolim, 2015). The “Incubus” type refers to feelings of chest pressure and shortness of breath. Cheyne et al. (1999) observed a correlation between types (a) and (c). Cheyne and Girard (2009) consider that out-of-body experiences and SP hallucinations have different neurobiological basis. The former would be caused by altered neural processing in temporoparietal cortex areas, which participate in the integration of visual, auditory, vestibular, and proprioceptive information to encode for body imagery and notion of self (Blanke et al., 2004, 2005; Jalal and Ramachandran, 2014), while the latter have been linked to abruptly going in and out of rapid-eye-movement sleep (REMS).

REMS is strongly associated with the vivid and richly emotional visual events experienced during sleep, which we call dreams (Aserinsky and Kleitman, 1953; Dement and Kleitman, 1957). REMS presents changes in vital signs such as blood pressure, respiratory rate and heart rate. Except for vital organs (e.g., heart and lung muscles) and genitals, the body as a whole is paralyzed during REMS. The absence of myographic activity is known as muscle atonia (Hobson et al., 2000). Muscle tonus decreasing during REMS occurs through the action of a descending inhibitory system from specific brainstem nuclei to the anterior column in the spinal cord, and then to muscles (Jouvet and Delorme, 1965). The main neurotransmitters associated with this network are GABA and glycine (Brooks and Peever, 2012). This system prevents animals from performing “in real life” the imaginary movements that they make when dreaming, which would render them extremely vulnerable, and therefore subject to be preyed upon. This idea derives mainly from the pioneering work of Jouvet (1979), who, upon damaging specific brainstem nuclei, noted that animals exhibited typical behavior such as running, cleaning themselves and masticating during REMS, as they no longer possessed muscle inhibition. These behavior patterns have been readily associated – hypothetically – to dreaming (Jouvet and Delorme, 1965; Jouvet, 1979).

Dahlitz and Parkes (1993) speculate that a lack of synchrony between changes in brain activity and muscle atonia is the mechanism responsible for bodily immobilization during SP. In general terms, during SP the brain falls back to an activity pattern similar to when individuals are awake; however, their muscles remain in the typical REMS atony, thus subjects feel as though they have awakened, and yet are unable to move (Dauvilliers et al., 2007; Nishino, 2007; Mahowald et al., 2011).

Despite sharing a few characteristics, there is an important dissimilarity between SP and dreams (Blackmore and Parker, 2002). When dreaming, we do not know that we are, in fact, dreaming, except in cases of lucid dreams (Van Eeden, 1913; Laberge et al., 1981; Erlacher and Schredl, 2008; Voss et al., 2009; Mota-Rolim and Araujo, 2013; Mota-Rolim et al., 2013; Dresler et al., 2014). On the other hand, during SP subjects know they were asleep but have woken up. In addition, SP experience is usually more aggressive than a normal dream, and there are four times more references to body parts (e.g., feelings of thorax pressure or paralyzed limbs) in the former relatively to the latter (Blackmore and Parker, 2002).

According to Hufford (2005), until recently SP was constantly underdiagnosed as a narcolepsy symptom. Narcolepsy is a disorder characterized by abnormalities in sleep regulation, including abrupt and involuntary sleep attacks associated with cataplexy (sudden loss of muscle tone), which usually follows a strong burst of emotion. Although it may be associated with Narcolepsy, SP can occur separately: the so-called isolated SP (American Academy of Sleep Medicine [AASM], 2014).

## Old, Ugly, and Ragged: Who is the *Pisadeira*?

Ferreira (1986) and Houaiss and Villar (2009) define the Pisadeira as a crone with long fingernails who lurks on roofs at night in order to trample on the chest of those who sleep. Luís da Câmara Cascudo - a renowned folklorist born in the Brazilian state of Rio Grande do Norte - adds further details to this physical description by mentioning Pisadeira’s gauntness and unkempt hair (Cascudo, 2012). He states that Pisadeira:

… is the nightmare personified in an old man or woman. The nightmare, or the roman “nocturna oppressio,” has always been explained by the evil intervention of an incubus, a demon or a malevolent spirit. In many cultures, the nightmare – also known as the classic oneirodynia, was due to a giant or a dwarf, a terrible woman or man that, taking advantage that one is sleeping, would sit upon their stomach and pressure their thorax, disturbing one’s breathing (p. 568).

Cascudo (2012) continues his description of Pisadeira looking for etymological references in other languages. He states that the word for nightmare in Portuguese, *pesadelo*, derives from “peso” or “pesado,” which means heavy; in French *cauchemar*, from the ancient verb *chaucer*, from Latin *calcare*, denotes to press or to push; finally, the English word *nightmare* is the night demon, or devil.

In the countryside of the Brazilian states of Minas Gerais and São Paulo, the Pisadeira possesses different physical features: she is a “fat” and “heavy” Afro-Brazilian adept at stepping on the abdomen of those who sleep on a full stomach or belly up. There is also a northeastern variant name for the Pisadeira around the São Francisco River, the so-called “*Pesadeira*.” She has the same features of her Minas Gerais and São Paulo state counterparts, except that the Pesadeira wears a red cap. The tale tells that if someone can scrounge it, Pesadeira loses her strength and will grant any wish in return for her cap (Lins, 1983). In Ceará state, this legend is called “*Pisador*” (Portuguese word for the one who steps on something/someone), and, differently from other regions, it is a masculine demon.

Cascudo (2012) believes that the Pisadeira is a direct descendant of the Portuguese myth known as “Fradinho da Mão Furada,” literally “Little Hand-Hole Friar.” It is said that Fradinho would enter people’s bedrooms, and place his “heavy hand” upon their chest, preventing them from screaming:

From Portugal, however, came the nightmare main elements. J. Leite de Vasconcelos, in his ‘Popular Traditions of Portugal,’ describes the origin of Pisadeira tormenting their hillbillies and rednecks. In Algarve, it is the “Little Hand-Hole Friar.” The Friar enters late at night in the alcoves, through the door keyhole. He has a red cap on his head, and straddles at ease upon the people, to assign them the worst nightmares. He goes away only when the person wakes up (p. 289). ‘..’ The nightmare is the devil, which has a cowl and a very heavy hand. When people sleep with the belly full, the nightmare puts his hand on the chest of the sleeper and leaves no possibility to shout (p. 290).’ From Portugal, of course, came the Pisadeira. But from where has Portugal received the ‘night oppressio?’ The influence of Provence on Portuguese land was long and powerful. Provencal people spread rhythms for the early verses. For Provencal culture, nightmare is an old woman, with the tricks of Pisadeira. Only in Provence and Portugal, she comes down the chimney, and goes to the sleeper chest (p. 569).

Cascudo (2012) also described an equivalent of Pisadeira for the native Brazilian Indians: “she was an old woman who, along with her procession of unspeakable agonies, would visit an individual” (p. 568). The native Brazilian tribe of the Tupi called her Kerepiiua. There was also the figure of the Jurupari, who, following catholic catechesis, was ascribed the meaning of “nocturnal demon,” its name a contraction of “i-ur-upá-ri” (she who comes to, or upon, the bed).

The Pires (2002) quotation below, taken from a dialog with a country bumpkin sitting at a bonfire, resembles descriptions of Pisadeira:

It’s a woman, this one, real skinny, got haseff’ dose real long, bony fingas with big ol’ nails! She got stubby legs, ratty hair, chin all pointed up and a nose real bent-like, bushy brows and eyes all a-glowin’... When, we finish our suppa an’ go sleep on our backs, she’ll come down from the roof an’ sit right on ya’chest, camberin’... camberin’ down... right on ya’belly! Now that’s why ya ought not let ya young-uns sleep on their backs (Pires, 2002, p. 89).

The Pisadeira also starred on the verses of Cora Coralina, a pseudonym of the Brazilian Goias-State poetess Anna Lins dos Guimarães Peixoto Brêtas (1889–1985). Despite her status as one of the greatest writers ever to grace Brazil, Cora Coralina had her very first book – *Poems from the Goias alleyways and other stories* – published only when she was 75 years old. In this book, Coralina makes the following reference to the *Pisadeira*:

That night it came to pass that Mrs. Jesuína was awakened by grunts, moans, almost, coming from the placemat. She scolded: “quiet, you imp, let us sleep...” All fell silent, and the night continued its spin in space and time. In the alcove, the yellow circle of the old oil lamp. The paintings of Saints, immovable on the walls. Then again, a renewed grunt and a few little moans, a thing of the underage. Once again uttered the old woman in all her nobility: “turn to the darn side, girl, that’s the Pisadeira, don’t you piss on that mat...” And then there was silence. The old woman went back to sleep, but woke up in the wee hours of the morning. “Jesuína, Jesuína.” No reply. She commented: “Tis how it goes, you fill up your breadbasket, the Pisadeira comes, won’t let you sleep, and in the morning you’re broken like hell” (Coralina, 2014, p. 32).

Unfortunately, Pisadeira – as well as other figures in Brazilian folklore, such as Saci and the headless Mule – is at a risk of being forgotten. Passed on from generation to generation in the Brazilian countryside, these oral traditions are losing their strength. One of the policies for the preservation of this heritage of Brazilian legends and myths is to give them their proper value (Moreira et al., 2009).

From what has been seen so far, it is possible to say that both the Pisadeira tale and SP share many features, such as a malevolent supernatural presence, feelings of chest pressure, difficulty to breath and to scream, and sleeping in supine position with full stomach. In the next sessions, we will review historical aspects of SP as well as its manifestation in other cultures.

## A Historical Outlook on Sleep Paralysis

Before the development of sleep science, there have been a series of magical and religious-based interpretations of SP throughout the history of human civilization, such as the Ancient Roman *nocturna oppressio* mentioned above (Cascudo, 2012). The first known description of SP comes from Hippocrates (~400 BC), and the Greeks named it 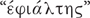 (ephialtes), roughly translated as “to pounce upon someone.” Artemidorus of Daldis (2nd century) in his book *Oneirocritica* – translated as “The Interpretation of Dreams” – associated *ephialtes* with the god Pan. The horned god of the woods and flocks could have sex with the dreamer during an *ephialtes*, and such was viewed as a promise of a great future income (Stewart, 2002).

This positive link with the sexual act would eventually change with the arrival of the Christian era of the Middle Ages. The *incubi* and the *succubi*, demons who sexually harassed their victims at night, would then emerge. The Latin name *incubus* would initially be a direct translation of *ephialtes*. However, it quickly acquired sexual connotations, given its proximity to *concumbere*, “to sleep (with),” and *concubinus*, a concubine. Much like the *incubus* was a male demon prone to abuse women, the *succubus* was a female demon who took advantage of men during sleep, and the origin of its name (*succubus*) means “to lie under.” Stewart (2002) comments that it was the Christianity that first negatively associated nightmares with erotic dreams, as its tenets were built upon the strict control of sexual instincts.

In early Enlightenment, with the beginning of scientific revolution, there is what appears to be the first medical description of an SP episode accompanied by hypnagogic hallucinations: Dr. Isbrand van Diemerbroeck (1609–1674) diagnosed a 50-year-old woman suffering from repeated spells caused by a *nightmare*, or incubus (Kompanje, 2008). Regarding the etymology of the word *nightmare*, Stewart (2002) describes its Scandinavian origins: *mare* comes from *mara*, a spirit that, in the Northern mythology, was said to torment or suffocate sleepers. The woman investigated by Diemerbroeck exhibited chest pressure, shortness of breath, inability to move, and an association of this experience with sleeping in the supine position (Kompanje, 2008), just like those visited by the Brazilian Pisadeira (Cascudo, 2012).

**FIGURE 1:**
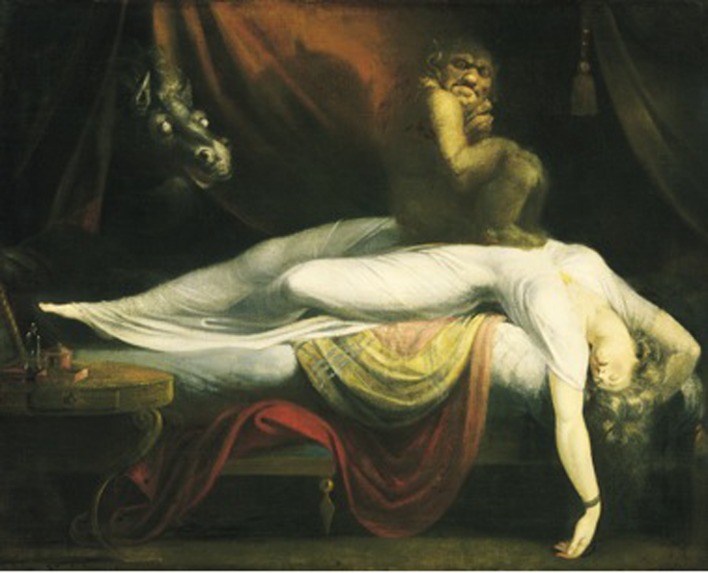
**The Nightmare (1871) by Henry Fuseli**.

There is a major source of confusion between the *nightmare* and SP. Before the 15th century, a “nightmare” was synonymous to spiritual attack, believed to be orchestrated by a witch. However, under the influence of Enlightenment, the “nightmare” became grouped under a larger category of “bad” dreams, and lost its specificity to SP characteristics (Hufford, 1982). Nevertheless, Henry Fuseli depicted the overlap between nightmare and SP in his most famous painting, “The Nightmare” (1781) (**Figure 1**). In this painting, an elf sits upon the chest of a lifeless-like woman draped in white over the end of a bed. In the upper left corner of the painting, just behind scarlet curtains, stands the ghostly head of a horse (Myrone, 2001). This painting has been interpreted as a “classical” pictorial representation of SP, of which the painter himself might have been victim, according to Kompanje (2008).

In the literary domain, Guy de Maupassant’s “Le Horla” is viewed by Cheyne (2001, 2015) as a case of SP. In the first version of the tale, Dr. Marrande introduce to his fellow scientists one of his patients, dubbed as “the strangest and most unsettling case” in his career as an alienist. The protagonist narrates his misfortunes from a first-person perspective, describing that an invisible being torments him every night. He is assaulted in his sleep by a “dreadful feeling of a crushing weight on my chest, and of a mouth that was eating up my life.” He blames his mysterious visitor – whom he names “Horla” – for his insomnia and loss of weight. In the final version of the tale, the narrator describes an episode in which, as he lies and sleeps, he also feels someone “approaching me, looking at me, feeling me, climbing into my bed, kneeling on my chest, taking my neck in his hands and squeezing” (Maupassant, 2009, p. 250), similarly to the Pisadeira (Cascudo, 2012). Curiously, the protagonist attributes his mental illness to a Brazilian ship he saw, believed to have spread an “epidemic madness,” in which people were possessed by a kind of vampire that feeds of their lives while they sleep.

### Contemporary SP Manifestation

A modern day retelling of SP would correspond to the so-called “alien abductions” (Shermer, 2011). Mack (1997) defines these cases as narratives (conscious or aided by hypnosis) describing the abduction by aliens, which are recorded in the absence of altered mental states – such as those induced by psychotropic substances. According to Shermer (2011), the appearance of aliens in the popular imagination took place after the supposed crash landing of an unidentified flying object in Roswell, New Mexico, in 1947. Shermer (2011) believes that the abduction narratives have their origin in a special broadcast by NBC in 1975, based on the extraordinary accounts by Barney and Betty Hill. The Hill couple described experiences that have become a standard for thousands of people who have experienced the same phenomenon: lights in the middle of the night, body paralysis, dissections, examinations, etc. The “classic” look of extraterrestrial kidnappers – bald, bigheaded humanoids with elongated eyes – was in fact a product of NBC artists. Following the dissemination of these accounts in newspapers, tabloids and TV programs, abduction reports rose exponentially. Shermer (2011) cites Strieber’s (1987) Communion and Mack’s (1997) Abduction as examples of literary landmarks that helped perpetuate the belief in the supposed veracity of alien abductions.

Clancy et al. (2002) and McNally et al. (2004) questioned the veracity of such reports – most of them extracted via hypnosis. According to McNally and Clancy (2005), human memory is malleable: false memories can be implanted by the suggestion of therapists. After conducting a survey with ten individuals that had been “abducted,” Clancy and McNally (2005) have found a substantial occurrence of SP episodes in this group compared to a control group. These authors additionally noted the similarity between alien abduction experiences and SP: immobility, feeling of a threating presence, feeling of levitation, flashing lights, bright objects, and other hallucinations.

## Sleep Paralysis in Other Cultures

Sleep paralysis represents a fairly strong evidence of how a given neurobiological phenomenon can be interpreted and shaped by different cultural contexts. Field studies conducted in many different parts of the world detected the same phenomenon occurring under a myriad of ethnic and religious perspectives. In these cases, the few seconds, or minutes, of an SP episode – a veritable mish-mash of terrifying sensations – give rise to supernatural interpretations (Hinton et al., 2005).

The closest language to Portuguese is Spanish; interestingly, SP in Mexico is known as “se me subió el muerto,” translated as “a dead body climbed on top of me” (for comprehensive review, see Sharpless and Doghramji, 2015). The idea of a “phantasmagoric weight” is also found in the Pisadeira tale. In Catalonia, the Pesanta is a black animal (usually a dog or a cat) that invades houses at night and steps over people’s chests, disturbing their breath and causing nightmares, similar to the Pisadeira. Curiously, both Pesanta and the Fradinho da Mão Furada (who originated the Pisadeira, as mentioned above) have holes in their hands.

Beyond Latin cultures, Kirmayer and Law (2005) studied the Inuit people, who are Eskimos living in Canada’s subarctic region. They refer to SP as *uqumangirniq*, an experience closely linked to the spiritual world, which courses with compromised motor functions (inability to move, talk, and/or scream), hallucinations, and the frightening manifestation of a shapeless, or faceless, presence. The Inuit believe that the *angakkuit* (shamans) are the main responsible for the *uqumangirniq*. Although the shamans exert a benign influence by organizing activities ranging from rites of passage to the interpretation of dreams, some shamans involved in power disputes cast spells (*ilisiiqsijuq*) on their opponents. One type of *ilisiiqsijuq* consists of attacking the enemy as they sleep, since at this moment the connection between body and soul (*tarniq*) is fragile. If a shaman can permanently separate the *tarniq* from its body, that individual dies.

In the Japanese tradition, the 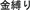 (*kanashibari*) is the cultural equivalent of SP (Fukuda et al., 1987). Translated as “the state of being totally bound, as if constrained by metal chains,” the *kanashibari* may emerge through the spell of a summoner, who uses a vengeful spirit to suffocate his enemies. The *kanashibari* is a popular phenomenon in the Far East, often represented in *mangas*, the Japanese comic books. Despite the fact that the phenomenology of SP in both Inuit and Japanese is very similar to the “Pisadeira,” they attribute to SP a human origin - from spells of shamans or summoners – which does not happen in the Brazilian tale.

Through interviews with one hundred Cambodian refugees in an American psychiatric clinic, Hinton et al. (2005a) observed a high incidence of SP among survivors of the Pol Pot dictatorial regime (1975–1979): 42% of the subjects reported at least one SP episode per year. These patients referred to SP as *khmaoch sângkât*, “the ghost that pushes you down.” According to them, a supernatural being, or a ghost, would put their hands on the chest or neck of the victim while sleeping in supine position, making it difficult to breathe. There are four ways the supernatural beings could look like: (i) a tall, black shadow lacking a defined outline; (ii) a red-eyed, canine-toothed being, dressed in a Khmer Rouge cloak who brandishes a knife or club; (iii) a simian-like demon; (iv) an *ap* – a grotesque creature embodied solely by the head of a woman and her bowels. Regarding the ghosts, when someone is killed violently, or buried without the proper funeral rites – something recurrent during the Pol Pot period – the spirit would be doomed to walk the Earth and haunt the living by showing them the afterlife state of penury.

The Asian immigrants from the Hinton et al. (2005b) sample exhibited high levels of post-traumatic stress disorder (PTSD). These subjects had certain physical SP symptoms associated with the severe, traumatic experiences they went through under the yoke of the Khmer Rouge. For example, they associated dyspnea with near-drowning experiences during monsoon periods typical to Southeast Asia, or with their witnessing executions in which victims wore a bag over their head. Chest pressure, a typical SP feature, was associated with the chest pain resulting from heavy loads they were forced to carry in rice farms run by their tormentors. The association of SP with PTSD in Cambodian people does not appear in the Pisadeira tale; however, there are no systematic studies of SP in the Brazilian society.

Sleep paralysis appears in many other cultures around the world with regional variances. In Thailand, the 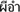 (*phi am*) is a ghost that haunts subjects when half-asleep and unable to move (Cassaniti and Luhrmann, 2011). Egyptians believe that SP is caused by the 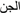 (*Jinn*), who are malevolent spirit-like creatures (Jalal and Hinton, 2013). Sharpless and Doghramji (2015) report that Ethiopians consider the 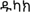 (*dukak*) an evil spirit that haunts the sleep. Similarly to the Brazilian Pisadeira, the Hmong people – an ethnic group from the mountain regions of Vietnam and Laos – believe that a “pressing spirit” sits on the chest of the sleepers and tries to asphyxiate them (Adler, 2011), while Chinese traditional people also believe that a type of “ghost oppression” causes SP (Yeung et al., 2005). Furthermore, the Yoruba people from Southwest Nigeria believe that the *Ogun Oru* is a female demon who possesses body and mind during dreaming (Aina and Famuyiwa, 2007), and in Newfoundland (a province of Canada) the *Old Hag* is a witch who sits on the sleeper (Firestone, 1985).

## Final Considerations

Sleep paralysis is characterized by body immobility, chest pressure, seeing scary figures, and/or feeling a frightening “presence,” which tend to happen during awakening in supine position. As described along this article, the interpretation of SP rebirths in different eras and cultures, such as the Greek “ephialtes” 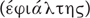, the *nocturna oppressio* of Ancient Roman, Fuseli’s “The nightmare,” the Japanese *kanashibari* (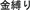), the Egyptian *Jinn* (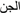), and the modern “alien abductions,” among others. Here, we report that SP in Brazil is usually described as Pisadeira attacks (Corso, 2002; Pires, 2002; Cascudo, 2012). Cascudo (2012) investigates Pisadeira etymology and relates it to the Portuguese word “pesadelo” (nightmare), akin to its original meaning, the Spanish *pesadilla* (“heavy” or “weighed”).

Since the Enlightenment, the “supernatural” experiences associated to SP have been interpreted as pathological. However, there are few connections between SP and other neuro-psychiatric disorders (Hufford, 2005) with the exception of Narcolepsy, as mentioned earlier (American Academy of Sleep Medicine [AASM], 2014). Yet, if SP episodes occur too frequently and/or intensely as to induce any physical, psychological or social suffering, the subject must be examined by sleep clinicians.

Noteworthy, there is a tendency to associate the Pisadeira and similar phenomena to a “superstition” believed by naïve, uneducated people. However, Hufford (2005) observed that a “spiritual” component of SP exists independently of social class or education level. Despite offering an alternative socio-biological interpretation to such episodes, here we do not intend to belittle this spiritual component. Instead, the goal of this work is to enrich the knowledge about these experiences and their psychological and cultural aspects.

## Author Contributions

All authors listed, have made substantial, direct and intellectual contribution to the work, and approved it for publication.

## Conflict of Interest Statement

The authors declare that the research was conducted in the absence of any commercial or financial relationships that could be construed as a potential conflict of interest.

## References

[B1] Adler S. R. (2011). *Sleep Paralysis: Night-Mares, Nocebos, and the Mind-Body Connection. New Brunswick, New Jersey, and*. London: Rutgers University Press.

[B2] Aina O. F. Famuyiwa O. O. (2007). Ogun Oru: a traditional explanation for nocturnal neuropsychiatric disturbances among the Yoruba of Southwest Nigeria. *Transcult. Psychiatry* 44 44–54. 10.1177/1363461507074968 17379609

[B3] American Academy of Sleep Medicine [AASM] (2014). *International Classification of Sleep Disorders: Diagnostic and Coding Manual*. Darien, CT: American Academy of Sleep Medicine.

[B4] Aserinsky E. Kleitman N. (1953). Regularly occuring periods of eye motility, and concomitant phenomena, during sleep. *Science* 118 273–274. 10.1126/science.118.3062.273 13089671

[B5] Blackmore S. J. Parker J. D. (2002). Comparing the content of sleep paralysis and dream reports. *Dreaming* 12 45–59. 10.1023/A:1013894522583

[B6] Blanke O. Landis T. Spinelli L. Seeck M. (2004). Out-of-body experience and autoscopy of neurological origin. *Brain* 127 243–258. 10.1093/brain/awh040 14662516

[B7] Blanke O. Mohr C. Michel C. M. Pascual-Leone A. Brugger P. Seeck M. (2005). Linking out-of-body experience and self processing to mental own-body imagery at the temporoparietal junction. *J. Neurosci.* 25 550–557. 10.1523/JNEUROSCI.2612-04.2005 15659590 PMC6725328

[B8] Brooks P. L. Peever J. H. (2012). Identification of the transmitter and receptor mechanisms responsible for REM sleep paralysis. *J. Neurosci.* 32 9785–9795. 10.1523/JNEUROSCI.0482-12.2012 22815493 PMC6621291

[B9] Burgess M. F. DuBreuil S. C. McNulty S. A. Pires M. Spanos N. P. (1995). The frequency and correlates of sleep paralysis in a university sample. *J. Res. Pers.* 29 285–305. 10.1006/jrpe.1995.1017

[B10] Cascudo L. C. (2012). *Dicionário do Folclore Brasileiro*. São Paulo: Global.

[B11] Cassaniti J. Luhrmann T. M. (2011). Encountering the supernatural - a phenomenological account of mind. *Relig. Soc.* 2 37–53.

[B12] Cheyne J. A. (2001). The ominous numinous: sensed presence and ‘Other’ hallucinations. *J. Conscious. Stud.* 8 133–150.

[B13] Cheyne J. A. (2015). Maupassants Der Horla und die kulturhistorische transformation des alien. *Z. Anomalistik Band* 15 S.235–259.

[B14] Cheyne J. A. Girard T. A. (2009). The body unbound: vestibular-motor hallucinations and out-of-body experiences. *Cortex* 45 201–215. 10.1016/j.cortex.2007.05.002 18621363

[B15] Cheyne J. A. Newby-Clark I. R. Rueffer S. D. (1999). Hypnagogic and hypnopompic hallucinations during sleep paralysis: neurological and cultural construction of the nightmare. *Conscious. Cogn.* 8 319–337. 10.1006/ccog.1999.0404 10487786

[B16] Chilcott L. Fukuda K. Ogilvie R. D. Takeuchi T. Vendittelli A. (1998). The prevalence of sleep paralysis among Canadian and Japanese college students. *Dreaming* 8 59–66. 10.1023/B:DREM.0000005896.68083.ae

[B17] Clancy S. A. Lenzeweger M. F. McNally R. J. Pitman R. K. Schacter D. L. (2002). Memory distortion in people reporting abduction by aliens. *J. Abnorm. Psychol.* 111 455–461. 10.1037/0021-843X.111.3.455 12150421

[B18] Clancy S. A. McNally R. J. (2005). Sleep paralysis, sexual abuse, and space alien abduction. *Transcult. Psychiatry* 42 113–122. 10.1177/1363461505050715 15881271

[B19] Coralina C. (2014). *Poemas Dos Becos De Goiás e Estórias Mais*. São Paulo: Global.

[B20] Corso M. (2002). *Monstruário: Inventário de Entidades Imaginárias e de Mitos Brasileiros*. Porto Alegre: Tomo Editorial.

[B21] Dahlitz M. Parkes J. D. (1993). Sleep paralysis. *Lancet* 341 406–407. 10.1016/0140-6736(93)92992-3 8094172

[B22] Dauvilliers Y. Arnulf I. Mignot E. (2007). Narcolepsy with cataplexy. *Lancet* 369 499–511. 10.1016/S0140-6736(07)60237-2 17292770

[B23] de Sá J. F. R. Mota-Rolim S. A. (2015). Experiências fora do corpo: aspectos históricos e neurocientíficos. *Cien. Cogn.* 20 189–198.

[B24] Dement W. C. Kleitman N. (1957). The relation of eye movements during sleep to dream activity: an objective method for the study of dreaming. *J. Exp. Psychol.* 53 339–346. 10.1037/h0048189 13428941

[B25] Dresler M. Eibl L. Fischer C. F. Wehrle R. Spoormaker V. I. Steiger A. (2014). Volitional components of consciousness vary across wakefulness, dreaming and lucid dreaming. *Front. Psychol.* 4:987 10.3389/fpsyg.2013.00987 PMC3877766 24427149

[B26] Erlacher D. Schredl M. (2008). Do REM (lucid) dreamed and executed actions share the same neural substrate? *Int. J. Dream Res.* 1 7–14.

[B27] Ferreira A. B. H. (1986). *Novo Dicionário Aurélio da Língua Portuguesa*. Rio de Janeiro: Nova Fronteira.

[B28] Firestone M. (1985). The “Old Hag”: sleep paralysis in Newfoundland. *J. Psychoanal. Anthropol.* 8 47–66.

[B29] Fukuda K. Miyasita A. Inugami M. Ishihara K. (1987). High prevalence of isolated sleep paralysis: kanashibari phenomenon in Japan. *Sleep* 10 279–286. 3629091 10.1093/sleep/10.3.279

[B30] Hinton D. E. Hufford D. J. Kirmayer L. J. (2005). Culture and sleep paralysis. *Transcult. Psychiatry* 42 5–10. 10.1177/1363461505050708 15881270

[B31] Hinton D. E. Pich V. Chhean D. Pollack M. H. (2005a). ‘The ghost pushes you down’: sleep paralysis-type panic attacks in a Khmer refugee population. *Transcult. Psychiatry* 42 46–77. 10.1177/1363461505050710 15881268

[B32] Hinton D. E. Pich V. Chhean D. Pollack M. H. McNally R. J. (2005b). Sleep paralysis among Cambodian refugees: association with PTSD diagnosis and severity. *Depress. Anxiety* 22 47–51. 10.1002/da.20084 16094659

[B33] Hobson J. A. Pace-Schott E. F. Stickgold R. (2000). Dreaming and the brain: toward a cognitive neuroscience of conscious states. *Behav. Brain Sci.* 23 793–842. 10.1017/S0140525X00003976 11515143

[B34] Houaiss A. Villar M. S. (2009). *Dicionário Houaiss da Língua Portuguesa*. Rio de Janeiro: Objetiva.

[B35] Hufford D. J. (1982). *The Terror That Comes in the Night: an Experience-Centered Approach to Supernatural Assault Traditions*. Philadelphia, PA: University of Pennsylvania Press.

[B36] Hufford D. J. (2005). Sleep paralysis as spiritual experience. *Transcult. Psychiatry* 42 11–45. 10.1177/1363461505050709 15881267

[B37] Jalal B. Hinton D. E. (2013). Rates and characteristics of sleep paralysis in the general population of Denmark and Egypt. *Cult. Med. Psychiatry* 37 534–548. 10.1007/s11013-013-9327-x 23884906

[B38] Jalal B. Ramachandran V. S. (2014). Sleep paralysis and the shadowy bedroom intruder: the role of the right superior parietal, phantom pain and projection of body image. *Med. Hypotheses* 83 755–757. 10.1016/j.mehy.2014.10.002 25459150

[B39] Jouvet M. (1979). What does a cat dream about? *Trends Neurosci.* 2 280–282. 10.1016/0166-2236(79)90110-3

[B40] Jouvet M. Delorme F. (1965). Locus ceruleus et sommeil paradoxal. *C. R. Seances Soc. Biol. Fil.* 159 895–899. 4221672

[B41] Kirmayer L. J. Law S. (2005). Inuit interpretations of sleep paralysis. *Transcult. Psychiatry* 42 93–112. 10.1177/1363461505050712 15881270

[B42] Kompanje E. J. O. (2008). ‘The devil lay upon her and held her down’: hypnagogic hallucinations and sleep paralysis described by the Dutch physician Isbrand van Diemerbroeck (1609-1674) in 1664. *J. Sleep Res.* 17 464–467. 10.1111/j.1365-2869.2008.00672.x 18691361

[B43] Laberge S. Nagel L. Dement W. C. Zarcone V. (1981). Lucid dream verified by volitional communication during REM sleep. *Percept. Mot. Skills* 52 727–732. 10.2466/pms.1981.52.3.727 24171230

[B44] Lins W. (1983). *O médio São Francisco: Uma Sociedade de Pastores Guerreiros*. São Paulo: Nacional.

[B45] Mack J. E. (1997). *Abduções*. Rio de Janeiro: EDUCARE.

[B46] Mahowald M. W. Cramer Bornemann M. A. Schenck C. H. S. (2011). Dissociation, human behavior, and consciousness. *Curr. Top. Med. Chem.* 11 2392–2402. 21906025 10.2174/156802611797470277

[B47] Maupassant G. (2009). *As Grandes Paixões: Contos de Guy de Maupassant*. Rio de Janeiro: Record.

[B48] McNally R. J. Clancy S. A. (2005). Sleep paralysis in adults reporting repressed, recovered, or continuous memories of childhood sexual abuse. *J. Anxiety Disord.* 19 595–602. 10.1016/j.janxdis.2004.05.003 15749576

[B49] McNally R. J. Lasko N. B. Clancy S. A. Macklin M. L. Pitman R. K. Orr S. P. (2004). Psychophysiological responding during script-driven imagery in people reporting abduction by space aliens. *Psychol. Sci.* 15 493–497. 10.1111/j.0956-7976.2004.00707.x 15200635

[B50] Moreira M. E. C. B. Consolmano R. A. C. Sobreira M. R. N. (2009). *Mitologia Brasileira: Resgate do Patrimônio Cultural. Anais da Jornada dos Cursos de História, Geografia e Arquitetura*, Vol. 1. Espaço, História e Globalização. Bauru: Universidade do Sagrado Coração.

[B51] Mota-Rolim S. A. Araujo J. F. (2013). Neurobiology and clinical implications of lucid dreaming. *Med. Hypotheses* 81 751–756. 10.1016/j.mehy.2013.04.049 23838126

[B52] Mota-Rolim S. A. Targino Z. H. Souza B. C. Blanco W. Araujo J. F. Ribeiro S. (2013). Dream characteristics in a Brazilian sample: an online survey focusing on lucid dreaming. *Front. Hum. Neurosci.* 7:836 10.3389/fnhum.2013.00836 PMC3857923 24368900

[B53] Myrone M. (2001). *Henry Fuseli.* Princeton: Princeton University Press.

[B54] Nishino S. (2007). Clinical and neurobiological aspects of narcolepsy. *Sleep Med.* 8 373–399. 10.1016/j.sleep.2007.03.008 17470414 PMC1978248

[B55] Pires C. (2002). *Conversas ao pé-do-fogo: Estudinhos – Costumes – Contos Anedotas – Cenas de Escravidão*. Itu: Ottoni.

[B56] Pires M. L. N. Benedito-Silva A. A. Mello M. T. Del Giglio S. Pompeia C. Tufik S. (2007). Sleep habits and complaints of adults in the city of São Paulo, Brazil, in 1987 and 1995. *Braz. J. Med. Biol. Res.* 40 1505–1515. 17934647

[B57] Sharpless B. A. Barber J. P. (2011). Lifetime prevalence rates of sleep paralysis: a systematic review. *Sleep Med. Rev.* 15 311–315. 10.1016/j.smrv.2011.01.007 21571556 PMC3156892

[B58] Sharpless B. A. Doghramji K. (2015). *Sleep Paralysis – Historical, Psychological and Medical Perspectives*. Oxford: Oxford University Press.

[B59] Sharpless B. A. McCarthy K. S. Chambless D. L. Milrod B. L. Khalsa S. R. Barber J. P. (2010). Isolated sleep paralysis and fearful isolated sleep paralysis in outpatients with panic attacks. *J. Clin. Psychol.* 66 1292–1306. 10.1002/jclp.20724 20715166 PMC3624974

[B60] Shermer M. (2011). *Por Que as Pessoas Acreditam em Coisas Estranhas: Pseudociência, Superstição e Outras Confusões dos Nossos Tempos*. São Paulo: JSN Editora.

[B61] Stewart C. (2002). Erotic dreams and nightmares from antiquity to the present. *J. Roy. Anthropol. Inst.* 8 279–309. 10.1111/1467-9655.00109

[B62] Strieber W. (1987). *Comunhão*. Rio de Janeiro: Editora Record.

[B63] Van Eeden F. (1913). A study of dreams. *PSPR* 26 431–461.

[B64] Voss U. Holzmann R. Tuin I. Hobson J. A. (2009). Lucid dreaming: a state of consciousness with features of both waking and non-lucid dreaming. *Sleep* 32 1191–1200. 19750924 10.1093/sleep/32.9.1191 PMC2737577

[B65] Wilson S. A. K. (1928). The narcolepsies. *Brain* 51 63–109. 10.1093/brain/51.1.63

[B66] Yeung A. Xu Y. Chang D. F. (2005). Prevalence and illness beliefs of sleep paralysis among chinese psychiatric patients in China and the United States. *Transcult. Psychiatry* 42 135–145. 10.1177/1363461505050725 15881273

